# Clinical Features of Adult Patients with Acute Hepatitis B Virus Infection Progressing to Chronic Infection

**DOI:** 10.1155/2014/358206

**Published:** 2014-10-02

**Authors:** Kojiro Michitaka, Atsushi Hiraoka, Yoshio Tokumoto, Keiko Ninomiya, Tomoyuki Ninomiya, Norio Horiike, Masanori Abe, Yoichi Hiasa

**Affiliations:** ^1^Gastroenterology Center, Ehime Prefectural Central Hospital, Kasugamachi 83, Matsuyama, Ehime 790-0024, Japan; ^2^Department of Gastroenterology and Metabology, Ehime University Graduate School of Medicine, Toon 791-0295, Japan; ^3^Department of Internal Medicine, Saiseikai Imabari Hospital, Imabari 794-0054, Japan

## Abstract

*Background*. Information regarding the progression of acute hepatitis B virus (HBV) infection to chronic infection in adults is scarce. *Methods*. Twenty-five adult patients with acute HBV infection (14 men and 11 women, 18–84 years old), whose clinical features progressed to those of chronic infection (group A) or did not (group B), were studied retrospectively. *Results*. There were 3 and 22 patients in groups A and B, respectively. Two of the 3 patients of group A lacked the typical symptoms of acute hepatitis. No differences were found between groups with respect to age, sex, or HBV genotypes. However, total bilirubin and alanine aminotransaminase levels were significantly lower in group A. *Conclusions*. Three of the 25 adult patients with acute HBV infection progressed to chronic infection. Hepatitis was mild in these patients. Patients with mild acute hepatitis B or unapparent HBV infection may have a higher risk of progressing to chronic infection.

## 1. Introduction

Hepatitis B virus (HBV) is a DNA virus with approximately 3200 base pairs. Approximately 350–400 million people are chronically infected with HBV and more than 3 billion people have been exposed to HBV worldwide [1, 2]. HBV induces a variety of liver diseases, ranging from acute or fulminant hepatitis to liver cirrhosis and hepatocellular carcinoma. HBV is one of the most important causes of liver cirrhosis and hepatocellular carcinoma [3]. On the other hand, hepatitis is self-limited in most adult patients with acute infection. Meanwhile 1-2% of patients progress to fulminant hepatic failure, and some progress to chronic infection. The rate of progression from acute to chronic HBV infection is reported to be 90% in newborns and 5–10% in adults [4, 5].

HBV can be classified into at least 8 genotypes with a divergence of more than 8% of nucleotide sequences [6–8]. There are some differences in clinical features and routes of transmission between genotypes [9, 10]. The rate of chronicity of genotype A infections is reported to be higher than those of other genotypes [11–14]. The progression of acute hepatitis B to chronic hepatitis is not rare in Western countries, but it is rare in Japan. The differences of the rates of chronicity of acute HBV infection supposed to be attributable to the different distribution of HBV genotypes; genotypes B and C are the predominant genotypes while genotype A was rare in Japan and common in Western countries. However, previous studies are based on the follow-up studies of apparent acute hepatitis B. The present study aimed to clarify the progression to chronic infection in adult patients with acute HBV infection including subclinical or unapparent patients who progressed to chronic infection.

## 2. Materials and Methods

### 2.1. Subjects

Among the 28 patients diagnosed with acute HBV infection who visited our hospital in the northwestern area of Shikoku Island, Japan, between 1998 and 2012, 25 survived without liver transplantation and were included in the present study.

### 2.2. Methods

HBsAg was assayed by chemiluminescence immunoassay (CLIA, Architect HBsAg QT, Abbott Japan, Tokyo, Japan) or reverse passive hemagglutination assay (Mycell II, Institute of Immunology, Tokyo, Japan). Anti-HBs was tested by CLIA (Architect AUSAB, Abbott Japan) or hemagglutination assay (Mycell II anti-HBs, Institute of Immunology). Hepatitis B e antigen (HBeAg), anti-HBe, and IgM-type antihepatitis B core (anti-HBc) were assayed by CLIA (Architect HBeAg, Architect HBeAb, Architect HBc-II, and Architect HBc-M, Abbott Japan, resp.). Anti-HBc was tested by CLIA (Architect HBc-II, Abbott Japan) or enzyme immunoassay (EIA, F-HBc, Sysmex, Kobe, Japan). HBV-DNA level was assayed using polymerase chain reaction (PCR, Amplicor HBV Monitor, Test, Roche Molecular Systems Inc., Pleasanton, USA) or real-time PCR, (AccuGene m-HBV Abbott Japan). HBV genotype was determined by serial invasive signal amplification reaction assay (Invader assay; BML Inc., Saitama, Japan) [15]. When the genotype could not be identified using this method, an EIA was performed (Immunis, HBV genotype EIA; Institute of Immunology Co., Ltd., Tokyo, Japan) [16]. Total bilirubin (T. bil) and alanine aminotransaminase (ALT) levels were examined every 1-2 weeks in the early stage of the disease. HBsAg was assayed every 1 or 2 months until negative results were yielded. The presence of human immunodeficiency virus (HIV) was assayed by the HIV antigen-antibody detection assay using a CLIA method.

The criteria of acute HBV infection were as follows: (1) positive for anti-HBc with a low titer (<10 by CLIA or <90% in 200-fold diluted serum by EIA) and positive for IgM-type anti-HBc or (2) positive for HBsAg in a previously HBsAg-negative patient. The criterion of progression to chronic infection was persistence of HBsAg from the onset of the disease for more than 6 months. Patients who lacked the typical symptoms or signs of acute hepatitis were deemed as having an unapparent infection.

The clinical features of subjects who progressed and did not progress to chronic infection were determined and analyzed. This study was retrospective in nature.

### 2.3. Statistical Analysis

Statistical analyses were performed using the *χ* ^2^ test, unpaired *t*-test, and Mann-Whitney *U*-test. The level of significance was set at *P* < 0.05.

## 3. Results

### 3.1. Comparison of Clinical Features

Among the 25 patients, 3 progressed to chronic infection while the other 22 did not. All 25 subjects were HIV negative. The clinical data of groups A and B are summarized in Table 1. No differences were found between groups with respect to age or sex. All subjects in group B and 1 subject in group A exhibited typical symptoms of acute hepatitis and were easily diagnosed with acute hepatitis at the early stage of the disease. Meanwhile, 2 subjects in group A lacked the typical symptoms of acute hepatitis (*P* < 0.01). Zero and 20 subjects in group A and group B, respectively, had T. bil >3 mg/dL (*P* < 0.01). Zero and 22 subjects in group A and group B had ALT >500 IU/L, respectively (*P* < 0.01). In addition, the average levels of T. bil and ALT were lower in group A.

Three of 3 and 10 of 22 subjects in groups A and B, respectively, were HBeAg-positive. HBV-DNA level was >7.0 log copies (LC)/mL in 3 of 3 but only 1 of 22 subjects in groups A and B, respectively (*P* < 0.01). Two and 1 subject in group A had HBV genotypes C and D, respectively. There was no difference in HBV genotypes between groups A and B.

### 3.2. Patients Progressing to Chronic Infection

**Figure 1:**
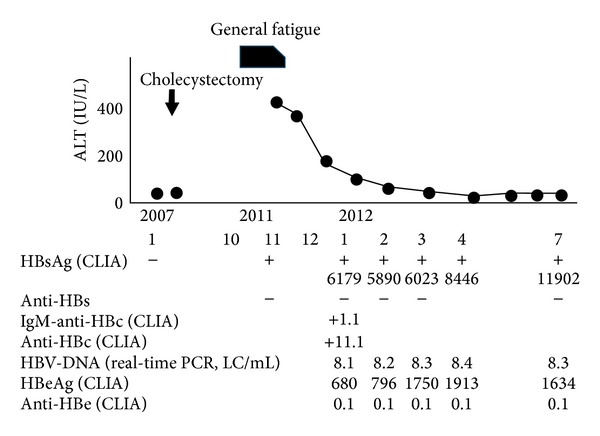
Clinical course of a patient infected with HBV genotype C who progressed to chronic infection (patient 1).

Patient 1 was an 84-year-old man who had chronic obstructive pulmonary disease and hypertension and was medicated by his home doctor. He had no history of liver disease and was negative for HBsAg in 2007. He had no family history of hepatitis B. He consulted his home doctor because of general fatigue in November 2011. He was not taking any medicine that may have suppressed the immune function. He was HBsAg-positive and presented with elevated ALT level (466 IU/L). He consulted our hospital in January 2012. He was positive for HBeAg and HBV-DNA. The cutoff indexes of IgM-type anti-HBc and anti-HBc (CLIA) were 1.1 and 11.1, respectively. His general fatigue improved within a few weeks. ALT levels improved gradually, reaching the normal range at 8 months after onset, and remained within the normal range thereafter. However, he was consistently positive for HBsAg, HBeAg, and HBV-DNA (Figure 1). He was infected with HBV genotype C.

Patient 2 was a 28-year-old woman. She had been a sexual worker since 2007. She complained about mild fatigue and consulted our hospital in 2008. Her ALT level was 44 IU/L and she was positive for HBsAg, HBeAg, and HBV-DNA. Her ALT level was within the normal range, and HBsAg and HBeAg were positive thereafter. HBV-DNA was consistently >8 LC/mL. She had been diagnosed with chronic hepatitis with mild activity or asymptomatic HBV carrier status (i.e., the immune-tolerant phase); therefore, IgM-type anti-HBc and anti-HBc were not tested in 2008. However, in 1999, it was proven later that she had been negative for HBsAg when she gave birth to her daughter. Although the daughter did not receive HBV vaccine after birth, she was negative for HBsAg and anti-HBc in 2010. The patient was suspected to have had acute HBV infection with mild hepatitis, which was considered an unapparent infection. She was infected with HBV genotype C.

Patient 3 was a 20-year-old woman. She was negative for HBsAg and HBV-DNA when she donated blood in 1998. She was HBsAg-positive and had an elevated ALT level when she donated again in 1999. She had a history of sexual contact with a man a few months before the donation in 1999. She and her sexual partner were found to be infected with HBV genotype D. She was HBsAg- and HBeAg-positive. IgM-type anti-HBc was positive (cutoff index value 5.4) and anti-HBc was also positive with a high titer (98% in 200-fold diluted serum). Liver biopsy prompted a histological diagnosis of chronic hepatitis with stage 1 and grade 2. She was treated with interferon for 1 month in 2000. She became HBsAg- and HBeAg-negative after the treatment. This patient was also suspected to have had unapparent acute HBV infection.

All of these 3 patients were not drug abusers and were not alcohol abusers. They did not take any medicine that may suppress immune functions. The details of the clinical courses and complete nucleotide sequences of HBV in patients 2 and 3 have been reported previously as case reports [17–19]. In patient 1, precore (nt 1986) and core promoter (nt 1762 and nt 1764) sequence of HBV-DNA were analyzed by polymerase chain reaction-enzyme-linked minisequence assay (PCR-ELMA) and PCR-scintillation proximity assay (PCR-SPA), respectively. He was infected with HBV without mutations in these positions.

## 4. Discussion

It must be noted that the present study was not aimed at knowing the rate of chronicity in acutely HBV-infected patients, because it is extremely difficult to collect the unapparent cases of acute HBV infection. The purpose of this study is to know the clinical features of patients with acute HBV infection who progressed to chronic infection.

All 3 patients in group A in the present study had been negative for HBsAg for at least 1 year before testing positive. Data regarding anti-HBc and HBV-DNA negativity before the onset of hepatitis were available in patient 3 but not in patient 1 or patient 2. Therefore, the possibility of HBV reactivation from HBsAg-negative carrier or resolved hepatitis status in these 2 patients cannot be excluded completely. However, these patients did not take any medicine that may suppress immune function, were not drug abusers or alcohol abusers, and were not in immune-suppressed state. Therefore, the risk of HBV reactivation is presumed to be very low.

Details of the clinical course and complete HBV genome sequences of patient 2 and patient 3 in the present study had been reported as case reports. Patient 2 was infected with genotype C HBV, with no mutations in core promoter (nt 1762, nt 1764) and precore (nt 1896). The case report indicated the possibility that acute infection of HBV genotype C infection in adult may progress to chronic infection, which had been reported to be rare in genotype C. Patient 3 was infected with genotype D HBV, and this HBV also had no mutations in core promoter (nt 1762, nt 1764) and precore (nt 1896). It has been shown in these case reports that the sequences of both strains were not very peculiar compared with many other strains of the same genotype in this district and specific mutations that related to chronicity of acute HBV infection had not been found.

Factors related to the severity of acute HBV infection and its clinical outcomes have been reported previously [20–22]. However, the factors related to the chronicity of HBV in acutely infected patients have not been fully elucidated. Both host and viral factors are suspected to affect the progression to chronic infection in acute HBV-infected patients [23–25]. The rate of chronicity in immunocompromised patients such as patients coinfected with HIV is reported to be high [24, 25]. In the present study, 3 of 25 adult patients with acute HBV infection progressed to chronic infection. These 3 patients were not compromised host. There were no differences between groups A and B with respect to age and sex, but T. bil and ALT levels were significantly lower in group A than those in group B. None of the 3 patients in group A had jaundice; their ALT levels were less than 500 IU/L, whereas HBV-DNA levels were high. Two of the 3 patients in group A lacked the typical symptoms of acute hepatitis. It is suspected that the progression from acute to chronic infection appears to represent a failure of immune clearance of virus-infected cells and it is marked by persistently high levels of HBV-DNA and HBeAg in serum. It has been described in a few literatures that the accompanying acute hepatitis is typically mild and subclinical with only modest serum ALT elevations and no jaundice in patients with acute hepatitis B who progressed to choric infection [5, 26]. The present data are concordant with these previous reports. Though the data of HBV sequences in the present study are not enough, all 3 patients of group A were infected with HBV without mutations in core promoter (nt 1762, nt 1764) and precore (nt 1896). It is well known that these mutations relate to severe hepatitis. It is needed to investigate further the relation of wild type HBV and chronicity of acute HBV infection in the future.

Among the 3 patients of group A, 2 and 1 were infected with genotypes C and D, respectively. There was no difference in HBV genotypes between groups A and B. These data indicate that acute infection with genotype C or D in adult can possibly progress to chronic infection in acutely infected adult patients, especially in those with mild acute hepatitis.

HBV genotype is a factor known to be related to the chronicity of acute HBV infection. The chronicity of genotype A is reported to be high, whereas that of genotype C is low; the rates of the chronicity of genotypes A and C infections are reported to be 3–23% and 0-1%, respectively [5–8]. Another report indicates that the chronicity of genotype D is supposed to be lower than that of genotype A [27]. The present results are not similar to the results of these reports in this respect. Many of these previous reports analyzed the follow-up data of patients presenting with clinical features of acute hepatitis. However, there is no report on the follow-up data of subclinical patients with acute HBV infection. Thus, the present study may indicate that the rate of chronicity in unapparent or subclinical cases of acute HBV infection with genotypes C and D might not be very low. However, it is difficult to study unapparent or subclinical cases of acute HBV infection. In the present study, 2 cases were unapparent infection, which were diagnosed as acute infection only because of accurate patient history. This highlights the importance of precisely taking the histories of patients and their families, even if they are supposedly chronically infected with HBV. The rate of chronicity of subclinical cases of acute HBV infection should be studied in greater detail in the near future.

In conclusion, adult patients with mild acute hepatitis B or subclinical HBV infection may have a higher possibility of progressing to chronic infection regardless of HBV genotypes.

## Figures and Tables

**Table 1:** Clinical data of the subjects according to HBV genotypes.

	Group A	Group B	*P*
Number of patients	3	22	N.S.
Male : female	1 : 2	13 : 9	N.S.
Age	28 (20–84)	35 (18–81)	N.S.
Apparent acute hepatitis	1 (33%)	22 (100%)	*P* < 0.05
Fulminant hepatitis	0	3 (13.6%)	N.S.
T. bil >3.0 mg/dL	0	20 (90.9%)	*P* < 0.01
T. bil (mg/dL), max	1.00 ± 0.36	8.43 ± 5.14	*P* < 0.05
ALT >500 IU/L	0	22 (100%)	*P* < 0.01
ALT (IU/L), max	250 ± 211	2733 ± 1540	*P* < 0.01
HBeAg (positive)	3 (100%)	10 (45.5%)	N.S.
HBV-DNA >7 LC/mL	3 (100%)	1 (4.5%)	*P* < 0.01
Genotype A : B : C : D	0 : 0 : 2 : 1	3 : 1 : 14 : 1 (undetermined 3)	N.S.

N.S.: not significant; T. bil: total bilirubin; ALT: alanine aminotransferase; LC: log copies; HBeAg: hepatitis B e antigen; HBV: hepatitis B virus.
